# Metabolomic biomarker differences of sarcopenia in older patients with sepsis

**DOI:** 10.3389/fmed.2026.1797866

**Published:** 2026-04-30

**Authors:** Yiyang Liu, Tianren He, Siyu Chen, Ziyi Li, Xue Mei, Xinhua He, Na Shang

**Affiliations:** 1 Beijing Key Laboratory of Cardiopulmonary-Cerebral Resuscitation Innovation and Translation, Beijing, China; 2 Beijing Chaoyang Hospital Emergency Medical Center Affiliated to Capital Medical University, Beijing, China

**Keywords:** biomarkers, elderly, metabolomics, sarcopenia, sepsis

## Abstract

**Background:**

Sarcopenia has a high incidence in older patients with sepsis and is closely associated with poor prognosis, but its metabolic characteristics under this specific stress state remain unclear. This study aims to reveal the specific plasma metabolomic changes related to sarcopenia in older patients with sepsis.

**Methods:**

A prospective observational cohort design was adopted, involving 84 older patients with sepsis, who were divided into a sarcopenia group (35 cases) and a non-sarcopenia group (49 cases). Fasting plasma samples were collected within 24 h, and untargeted metabolomic analysis was performed using ultra-performance liquid chromatography-tandem mass spectrometry (UPLC-MS/MS) technology. Multivariate statistical methods such as principal component analysis (PCA) and orthogonal partial least squares-discriminant analysis (OPLS-DA) were used to screen differential metabolites, and KEGG pathway enrichment analysis was conducted.

**Results:**

A total of 4,752 metabolites were identified, and 203 significantly differential metabolites were screened out (71 upregulated and 132 downregulated), indicating that the plasma metabolic profile of sarcopenic patients presents an “inhibitory” feature. The differential metabolites were mainly enriched in core pathways such as valine/leucine/isoleucine degradation, arachidonic acid metabolism, and steroid hormone synthesis. Specifically, metabolic pathways promoting muscle synthesis (such as steroid hormone and branched-chain amino acid metabolism) were generally downregulated, while pathways representing catabolism and injury (such as inflammatory mediator production) were significantly upregulated. Correlation analysis of differential metabolites revealed an interconnected metabolic disorder network.

**Conclusion:**

This study preliminarily reveals the unique plasma metabolic profile of elderly patients with sepsis complicated by sarcopenia, which is characterized by the impact on core pathways such as branched-chain amino acid degradation, steroid hormone synthesis inhibition, and arachidonic acid metabolism activation. These findings systematically depict the potential metabolic imbalance networks associated with this disease state, provide new clues for understanding its pathophysiological mechanisms, and offer an important theoretical basis and potential targets for the future development of early diagnostic biomarkers and targeted intervention strategies for this disease.

## 1 Introduction

Sarcopenia is a geriatric syndrome characterized by “reduced skeletal muscle mass, decreased muscle strength, and impaired muscle function” (1). It not only affects patients’ mobility and increases the risk of falls and fractures but also is closely associated with various adverse outcomes such as metabolic disorders, cardiovascular diseases, and tumors (2). It has become a non-negligible public health problem in the global aging society (3, 4). In older patients with sepsis, the incidence of sarcopenia is even higher, and it is closely related to worse clinical prognosis (5–7), but its metabolic characteristics under the specific stress state of sepsis remain unclear. As a core method for studying biological phenotypes, metabolomics can accurately capture subtle changes in the body’s metabolic profile (8–10). Skeletal muscle is the core organ of body metabolism, and the reduction in muscle mass and functional abnormalities in sarcopenic patients are inevitably accompanied by the remodeling of metabolic networks (11–13). As the core carrier for metabolite transport, plasma metabolomic profiles can directly reflect sarcopenia-related metabolic disorders (14, 15). Therefore, this study took elderly sepsis patients as the research objects, compared the plasma metabolic profiles between sarcopenic and non-sarcopenic patients through untargeted metabolomic analysis, screened differential metabolites between the two groups, analyzed differential metabolic pathways, further clarified the metabolic disorder characteristics related to sarcopenia in elderly sepsis patients, explored potential diagnostic biomarkers, and provided new evidence for early screening and disease assessment.

## 2 Materials and methods

### 2.1 General information

#### 2.1.1 Research objects

This was a prospective observational cohort study conducted in the Emergency Department of Beijing Chaoyang Hospital, Capital Medical University. A total of 84 patients with sarcopenia (35 cases) and non-sarcopenia (49 cases) admitted from January 2022 to November 2022 were included. To adjust for potential confounding effects of age, BMI, and activities of daily living (ADL) scores on metabolomic findings, these variables were included as covariates in univariate analysis of covariance (ANCOVA) during differential metabolite screening, thereby identifying metabolic features independently associated with sarcopenia. Other baseline characteristics, such as gender, showed no significant differences between the two groups (*p* > 0.05). Meanwhile, clinical indicators reflecting the severity of sepsis, such as Sequential Organ Failure Assessment (SOFA) score, Acute Physiology and Chronic Health Evaluation II (APACHE-II) score, Nutrition Risk in the Critically Ill (NUTRIC) score, the presence of septic shock, and levels of inflammatory indicators, also showed no significant differences between the groups (*p* > 0.05), indicating that the two groups of patients were comparable (Table 1).

**Table 1:** Comparison of baseline characteristics between the sarcopenia and non-sarcopenia groups.

Variable	Sarcopenia group (*n* = 35)	Non-sarcopenia group (*n* = 49)	*p*-value
Demographics			
Age, year, median (Q_L_, Q_U_)	85 (82, 90)	78 (71, 84)	0.0002
Gender			0.7962
Female (*n*, %)	16 (46)	25 (51)	
Male (*n*, %)	19 (54)	24 (49)	
BMI, kg/m^2^, mean (SD)	20.33 (2.93)	23.79 (3.67)	<0.0001
Clinical scores			
ADL, scores, mean (SD)	22 (33)	47 (39)	0.0012
GCS, scores, median (Q_L_, Q_U_)	10 (5, 14)	15 (7, 15)	0.0208
SOFA, scores, median (Q_L_, Q_U_)	6 (4, 8)	6 (4, 7)	0.6872
CCI, scores, median (Q_L_, Q_U_)	5 (5, 7)	6 (5, 8)	0.6843
SAPS, scores, median (Q_L_, Q_U_)	53 (39, 59)	41 (33, 55)	0.0532
CFS, scores, mean (SD)	7 (1)	6 (1)	0.0643
APACHE II, scores, median (Q_L_, Q_U_)	20 (16, 25)	15 (11, 22)	0.1601
NUTRIC, scores, median (Q_L_, Q_U_)	7 (6, 8)	6 (5, 8)	0.1367
Vital signs & support			
Invasive mechanical ventilation (*n*, %)	14 (40.00%)	24 (49.00%)	0.5533
CRRT (*n*, %)	3 (8.60%)	4 (8.20%)	1.0000
Vasopressor use (*n*, %)	19 (54.30%)	21 (42.90%)	0.4166
Temperature, °C, median (Q_L_, Q_U_)	36.5 (36.5, 38.0)	36.6 (36.2, 37.9)	0.5901
Heart rate, min, mean (SD)	100 (21)	100 (21)	0.9158
Respiratory rate, min, median (Q_L_, Q_U_)	18 (16, 20)	18 (17, 22)	0.4124
SBP, mmHg, mean (SD)	131 (33)	127 (31)	0.5371
DBP, mmHg, mean (SD)	69 (19)	72 (18)	0.4866
SpO_2_, %, median (Q_L_, Q_U_)	96 (88, 98)	94 (86, 96)	0.1560
FiO_2_, %, median (Q_L_, Q_U_)	0.3 (0.2, 0.3)	0.3 (0.2, 0.4)	0.2439
Laboratory values			
WBC, ×10^9^/L, median (Q_L_, Q_U_)	11.41 (8.42, 16.81)	9.87 (7.76, 14.58)	0.3862
Neutrophil, ×10^9^/L, median (Q_L_, Q_U_)	9.94 (7.16, 15.31)	8.32 (6.26, 14.46)	0.4352
Lymphocyte, ×10^9^/L, median (Q_L_, Q_U_)	0.90 (0.61, 1.26)	0.78 (0.54, 1.18)	0.2904
RBC, ×10^9^/L, median (Q_L_, Q_U_)	3.80 (3.30, 4.04)	3.76 (3.09–4.20)	0.8882
LDH, /L, median (Q_L_, Q_U_)	220.00 (191.50, 266.50)	244.00 (186.00, 340.00)	0.3116
BUN, mol/L, median (Q_L_, Q_U_)	9.47 (7.41, 16.32)	10.84 (7.46, 19.10)	0.6864
Creatinine, mol/L, median (Q_L_, Q_U_)	92.80 (55.10, 141.45)	103.60 (78.70, 171.40)	0.1883
PCT, g/L, mean (SD)	7.89 (18.03)	7.64 (15.09)	0.0960
CRP, g/L, median (Q_L_, Q_U_)	34.50 (21.80, 78.00)	34.50 (9.00–63.00)	0.2640
TnI, g/L, median (Q_L_, Q_U_)	0.09 (0.04, 0.21)	0.08 (0.03, 0.32)	0.9818

Data are presented as *n* (%) for categorical variables and mean ± standard deviation for continuous variables. *p*-values in bold indicate statistical significance (commonly at *p* < 0.05).

Q_L_, lower quartile; Q_U_, upper quartile; SD, standard deviation; BMI, body mass index; ADL, activities of daily living; GCS, Glasgow Coma Scale; SOFA, Sequential Organ Failure Assessment; CCI, Charlson Comorbidity Index; SAPS, Simplified Acute Physiology Score; CFS, Clinical Frailty Scale; APACHE, Acute Physiology and Chronic Health Evaluation; CRRT, continuous renal replacement therapy; BP, blood pressure; FiO₂, fraction of inspired oxygen; WBC, white blood cell; RBC, red blood cell; LDH, lactate dehydrogenase; BUN, blood urea nitrogen; PCT, procalcitonin; CRP, C-reactive protein; TnI, Troponin I.

#### 2.1.2 Inclusion criteria

(1) Diagnosis based on the 2019 Asian Sarcopenia Guidelines (16, 17): ① Low muscle mass (Lumbar 3 skeletal muscle index (L3 SMI): male <37.9 cm^2^; female <28.6 cm^2^); ② Low grip strength (male <28.0 kg; female <18.0 kg) (18, 19). (2) Hospital stay >24 h, age ≥65 years, and complete clinical data; (3) Able to cooperate with sample collection and clinical indicator detection; (4) Voluntarily participate in this study and sign the informed consent form.

#### 2.1.3 Exclusion criteria

(1) Malignant tumors (confirmed by pathological biopsy or imaging); (2) Severe endocrine diseases (such as diabetic ketoacidosis, thyroid storm); (3) Major surgical history within the past 3 months (such as orthopedic surgery, major abdominal surgery); (4) Long-term use of drugs affecting metabolism (such as glucocorticoids, immunosuppressants, long-term hypoglycemic/lipid-lowering drugs); (5) Cognitive impairment (MMSE score <24 points) unable to cooperate with the study.

### 2.2 Research methods

#### 2.2.1 Sample collection and processing

Fasting venous blood was collected from the subjects in the early morning, and plasma was separated by centrifugation and stored at −80 °C in an ultra-low temperature freezer. Standard protein precipitation extraction was performed before detection. Subsequently, untargeted metabolomic analysis was carried out using ultra-performance liquid chromatography-tandem mass spectrometry (UPLC-MS/MS) technology. To minimize the interference of therapeutic drugs on metabolomics results, the following strategies were implemented: (1) All blood samples were collected within 24 h after admission; (2) All patients received guideline-standard treatment to ensure no significant between-group differences in main medications; (3) Peaks corresponding to drugs and related exogenous compounds were actively excluded during metabolite annotation; (4) The orthogonal partial least squares-discriminant analysis (OPLS-DA) model was applied to remove variation unrelated to group assignment.

#### 2.2.2 Data analysis

The obtained raw mass spectrometry data were first subjected to preprocessing such as peak extraction, alignment, and calibration, and metabolite identification was performed by combining multiple databases. For statistical analysis, unsupervised principal component analysis (PCA) was first used to evaluate data quality and the natural separation trend between groups, then supervised orthogonal partial least squares-discriminant analysis (OPLS-DA) model was used to maximize the differences between groups, and the validity of the model was strictly verified. Differential metabolites were screened based on variable importance in projection (VIP) >1 and Student’s *t*-test *p* < 0.05, with further false discovery rate (FDR) correction using the Benjamini–Hochberg method, setting FDR < 0.05 as the significance threshold. Subsequently, KEGG pathway enrichment analysis was performed on these differential metabolites to explain their biological functions. Meanwhile, the diagnostic value of key metabolites was evaluated by receiver operating characteristic (ROC) curve.

#### 2.2.3 Software and tools

All data analyses were completed using R software (version 4.1.2) and related packages, including base package (for PCA and Pearson correlation coefficient calculation), MetaboAnalystR (version 1.0.1, for OPLS-DA analysis), ComplexHeatmap (version 2.9.4, for heatmap drawing), corrplot (version 0.92, for correlation plot drawing), fmsb (version 0.7.1, for radar plot drawing), igraph (version 1.2.11) and ggraph (version 2.0.5, for chord diagram and correlation network drawing), FELLA (version 1.2.0, for regulatory network construction).

## 3 Research results

### 3.1 Quality assessment of plasma metabolomic data

#### 3.1.1 Repeatability verification of quality control samples

**Figure 1:**
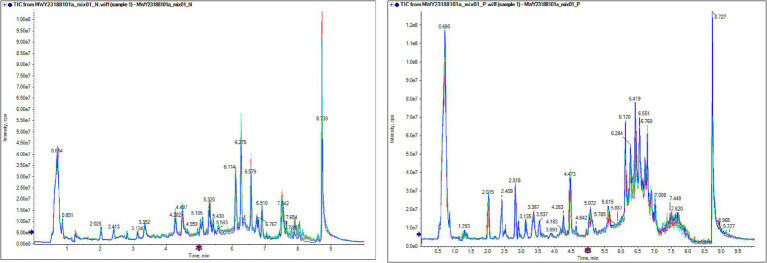
Overlay plot of TIC for QC sample mass spectrometry detection. The results show that the curves of the total ion current for metabolite detection have a high degree of overlap, that is, the retention time and peak intensity are consistent, indicating that the signal stability is good when the mass spectrometer detects the same sample at different times. The high stability of the instrument provides an important guarantee for the repeatability and reliability of the data.

**Figure 2:**
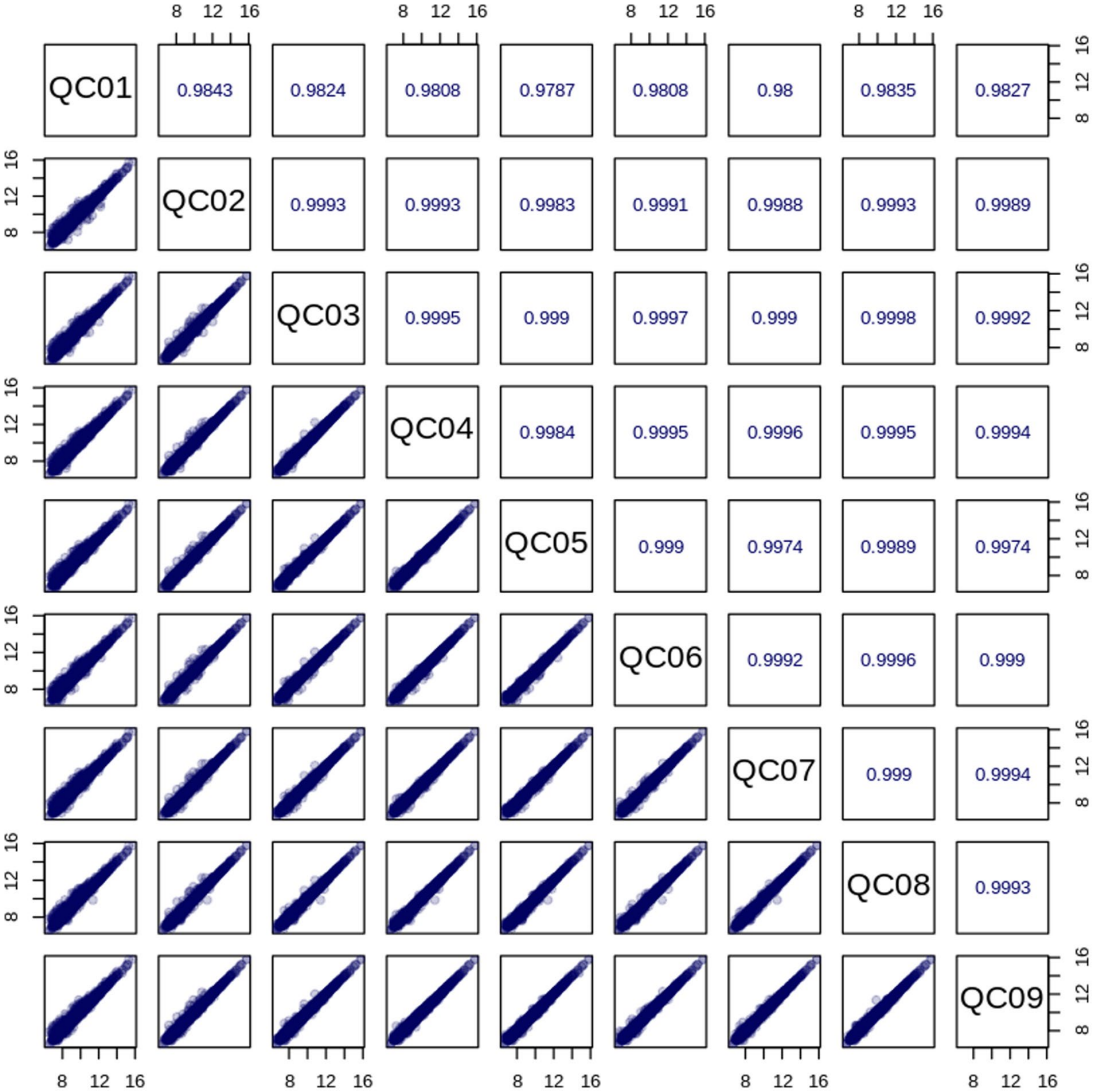
Correlation plot of QC samples. The diagonal squares represent the names of QC samples; the squares in the lower left corner of the diagonal are the correlation scatter plots of the corresponding QC samples, with the horizontal and vertical coordinates representing the metabolite content (after log processing), and each point in the figure represents a metabolite; the squares in the upper right corner of the diagonal are the Pearson correlation coefficients of the corresponding QC samples.

Quality control (QC) samples were prepared by mixing extracts of all research samples to evaluate the repeatability of the experimental process. Under both ESI^+^ and ESI^−^ ionization modes, the total ion current (TIC) diagrams of QC samples showed a high degree of overlap, with good consistency in retention time and peak intensity (Figure 1), indicating that the technical repeatability of metabolite extraction, separation, and detection processes was reliable. Pearson correlation analysis showed that the correlation coefficients |r| between QC samples were all close to 1 (Figure 2), suggesting strong stability of the entire detection system and small data fluctuation. Stability analysis of internal standard substances with known concentrations added to QC samples showed that their coefficients of variation (CV) were all less than 0.1 (range 0.0523–0.0743) (Supplementary Table 1), further confirming the accuracy of the detection process. Analysis of the CV value distribution of each group of samples by empirical cumulative distribution function (ECDF) found that the proportion of metabolites with CV <0.3 in QC samples exceeded 75% (Supplementary Figure 1), which met the strict requirements for data stability in metabolomic experiments and verified the scientificity and reliability of the experimental method in this study.

#### 3.1.2 Investigation of blank sample contamination

The extracted ion current (EIC) diagram of blank samples (containing only extract without plasma components) showed that there were no obvious signal peaks at the retention time corresponding to internal standard substances, only random noise peaks (Supplementary Figure 2), indicating that there was no obvious cross-contamination during sample processing, injection, and detection. The experimental environment and operation process were in line with specifications, excluding the interference of exogenous contamination on the experimental results.

### 3.2 Analysis of overall metabolomic differences between sarcopenic and non-sarcopenic patients

#### 3.2.1 Results of principal component analysis

Unsupervised PCA was used for dimensionality reduction of metabolomic data of all samples (including QC samples) to evaluate the natural clustering trend between groups and data reliability. The 2D and 3D PCA score plots (Supplementary Figures 3–5) showed that the samples of the sarcopenia group and the non-sarcopenia group presented an obvious separation trend in the principal component space, with good aggregation within the groups and no significant outliers, suggesting that there were essential differences in the plasma metabolic profiles between the two groups. The cumulative explanatory rate of the first 5 principal components reached 42.3%, among which the explanatory rate of the first principal component (PC1) was 11.08% and that of the second principal component (PC2) was 8.75% (Supplementary Figure 6), indicating that the extracted principal components could effectively capture the core information of the original data. Monitoring the instrument status through the PC1 control chart showed that the PC1 scores of QC samples fluctuated within ±3 standard deviations (Supplementary Figure 7), confirming that the instrument operated stably during the entire detection process without obvious drift, and the data quality was reliable.

#### 3.2.2 Results of orthogonal partial least squares-discriminant analysis

**Figure 3:**
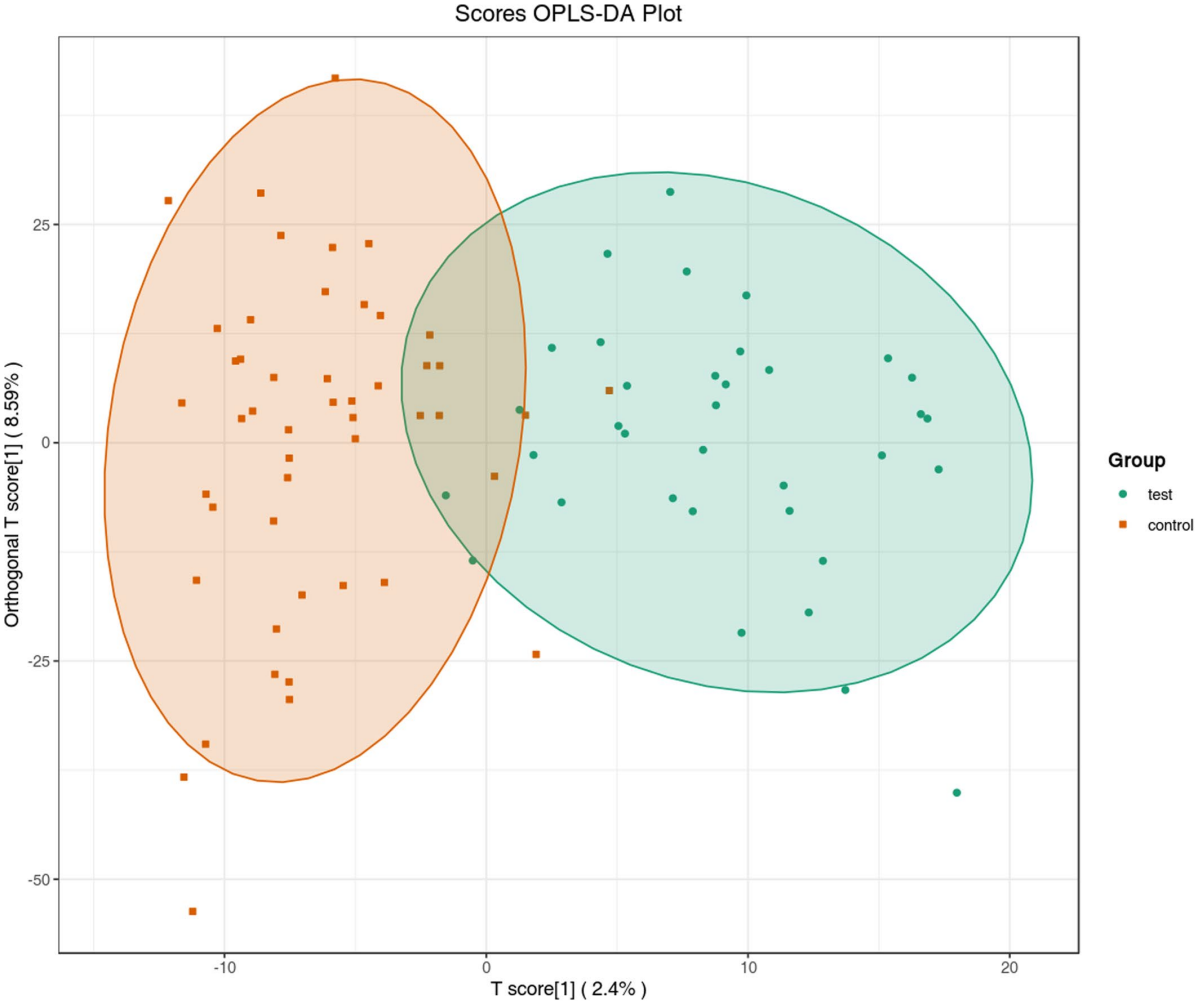
OPLS-DA score plot. The horizontal coordinate represents the predicted component score value, and the difference between groups can be seen in the horizontal direction; the vertical coordinate represents the orthogonal component score value, and the difference within the group can be seen in the vertical direction; the percentage represents the explanatory rate of the component to the data set.

To maximize the differences between groups and accurately screen differential metabolites, a supervised OPLS-DA model was used for further data analysis. The OPLS-DA score plot (Figure 3) showed that the samples of the sarcopenia group and the non-sarcopenia group achieved complete separation without cross-overlap, indicating that the model could effectively distinguish the metabolic profile characteristics of the two groups. The model verification results showed that *R*^2^*X* = 0.892, *R*^2^*Y* = 0.876, *Q*^2^ = 0.783. All three indicators were close to 1 and *Q*^2^ > 0.5, suggesting that the model had good explanatory power and predictive ability for the data; the results of 200 permutation tests showed *p* < 0.05, proving that the model had no overfitting phenomenon and was stable and reliable. After adjusting for confounding factors such as age, BMI, and ADL scores, the OPLS-DA model remained capable of significantly differentiating between individuals with sarcopenia and those without, suggesting that the observed metabolic alterations are independently associated with sarcopenia. The distribution characteristics of differential metabolites were analyzed by the S-plot diagram (Supplementary Figure 8). The horizontal coordinate was the covariance between metabolites and the principal component, and the vertical coordinate was the correlation coefficient. Metabolites close to the upper right and lower left corners had significant differences, among which red dots represented core differential metabolites with VIP >1, and green dots represented non-significant differential metabolites with VIP ≤1, providing clear targets for the subsequent screening of differential metabolites.

### 3.3 Screening and characterization of differential metabolites

#### 3.3.1 Results of differential metabolite screening

A total of 4,752 metabolites were identified in this study. Using the screening criteria of “VIP >1 and *p* < 0.05 (Student’s *t*-test)”, a total of 203 differential metabolites were screened out, including 71 upregulated metabolites and 132 downregulated metabolites (Table 2), indicating that the plasma metabolic profile of sarcopenic patients presented an obvious “inhibitory” feature, and most metabolic pathways had a downward trend.

**Table 2:** Statistics of the number of differential metabolites.

Group	Total	Down	Up
Sarcopenia group vs. Non-sarcopenia group	203	132	71

#### 3.3.2 Expression characteristics of differential metabolites

Through the calculation and sorting of fold change (FC), a dynamic distribution diagram of metabolite content differences was drawn, which intuitively showed the overall difference trend of metabolites between groups, with the top up-regulated and down-regulated metabolites highlighted. Typical upregulated differential metabolites included 9S-HOTrE (inflammation-related metabolite, FC = 2.3), L-arabitol (energy metabolism-related, FC = 2.1), etc.; typical downregulated differential metabolites included 1,4-androstadiene-3,17-dione (precursor of steroid hormone synthesis, FC = 0.35), D-glutamic acid (substrate for amino acid metabolism, raw material for muscle protein synthesis, FC = 0.42), etc. (Table 3).

**Table 3:** Results of differential metabolite screening.

Index	Compounds	Type
HMDB0247705	9S-HOTrE	Up
HMDB0003422	1,4-Androstadiene-3,17-dione	Down
HMDB0001873	Isobutyric acid	Down
HMDB0001851	L-Arabitol	Up
HMDB0000488	4E,15z-Bilirubin IXa	Down
HMDB0003339	D-Glutamic acid	Down

#### 3.3.3 Traceability analysis of differential metabolites

Traceability analysis of differential metabolites was performed through the KEGG database to clarify their sources and metabolic functions. The results showed that the sources of differential metabolites were diverse, including human endogenous metabolites (such as 9S-HOTrE, D-glutamic acid), microbial metabolites (such as 1,4-androstadiene-3,17-dione), etc., providing a new direction for in-depth exploration of the pathogenesis of sarcopenia.

### 3.4 Functional annotation and enrichment analysis of differential metabolites

#### 3.4.1 KEGG pathway annotation and enrichment analysis

**Figure 4:**
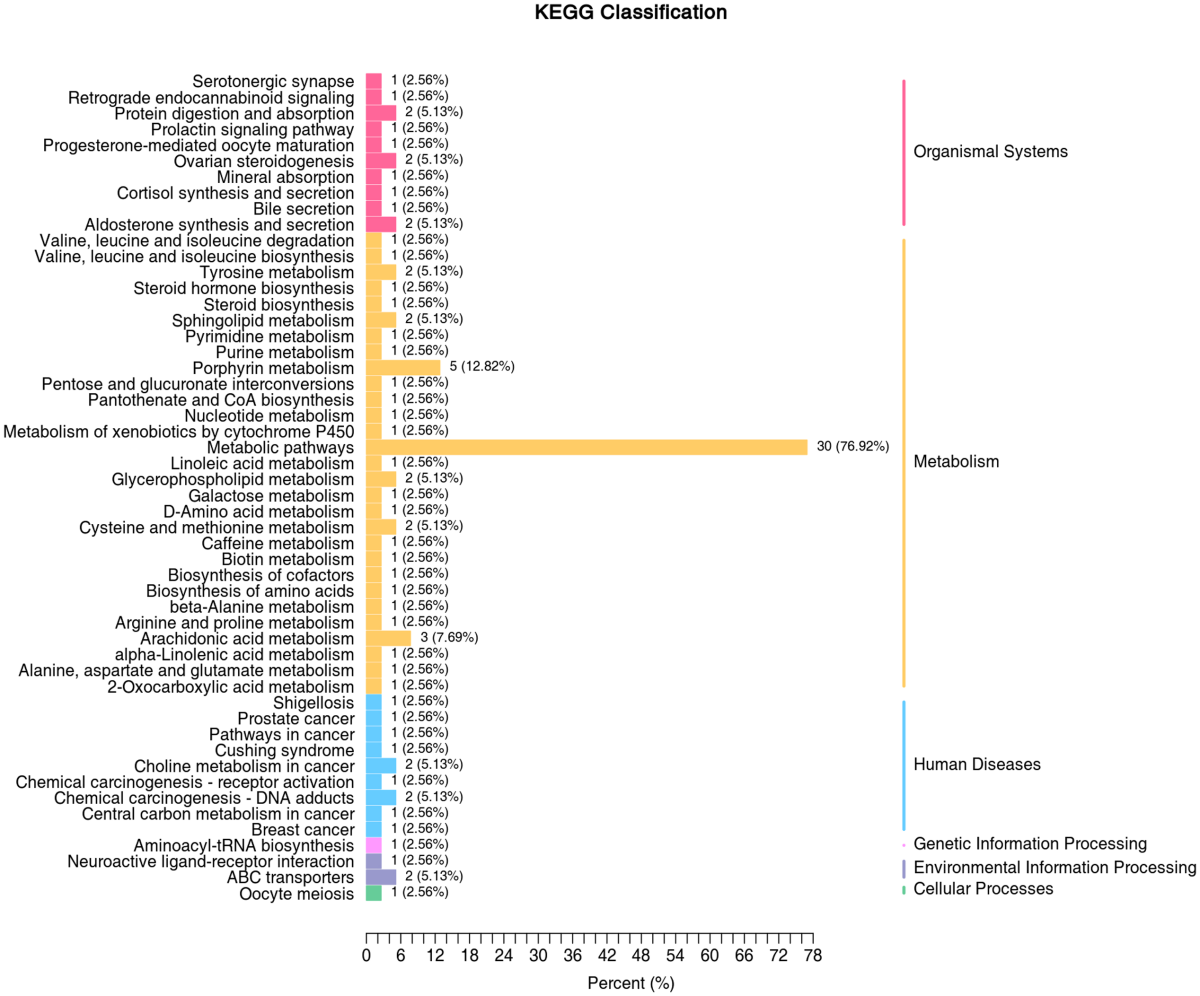
KEGG classification diagram of differential metabolites. The vertical coordinate is the name of the KEGG metabolic pathway, and the horizontal coordinate is the number of differential metabolites annotated to the pathway and the proportion of the number to the total number of annotated metabolites.

**Figure 5:**
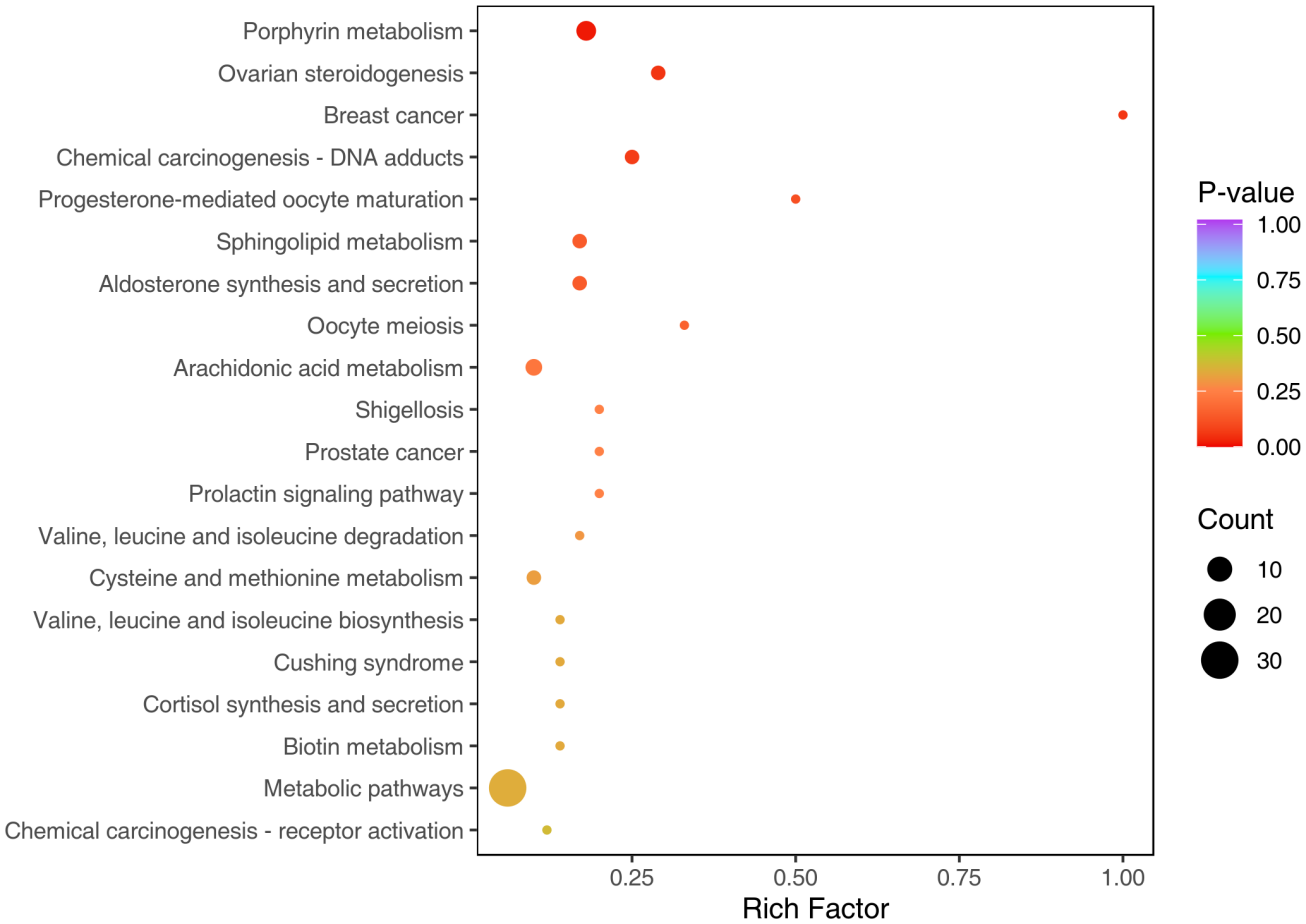
KEGG enrichment diagram of differential metabolites. The horizontal coordinate represents the rich factor corresponding to each pathway, the vertical coordinate is the pathway name (sorted by *p*-value), the color of the dot is the *p*-value, the redder the color, the more significant the enrichment. The size of the dot represents the number of differential metabolites enriched.

Pathway annotation and enrichment analysis of 203 differential metabolites were performed using the KEGG database, and 15 pathways were significantly enriched (*p* < 0.05) (Supplementary Table 2). They mainly included valine/leucine/isoleucine metabolism, cysteine/methionine metabolism, arachidonic acid metabolism, porphyrin metabolism, steroid hormone synthesis pathway, prolactin signaling pathway, Cushing’s syndrome-related pathway, etc. (Figures 4, 5). Rich factor analysis showed that the valine/leucine/isoleucine degradation pathway had the highest rich factor (0.87), followed by the arachidonic acid metabolism pathway (0.75), indicating that these pathways had the highest enrichment degree of differential metabolites and the closest association with sarcopenia.

Differential abundance (DA) score analysis showed (Supplementary Figure 9) that the steroid hormone synthesis pathway and amino acid degradation pathway were generally downregulated (DA score <0), and the energy metabolism-related pathways were partially upregulated (DA score >0). The results of KEGG pathway map annotation (Supplementary Figure 10) showed that inflammation-related metabolites (such as 9S-HOTrE) in the arachidonic acid metabolism pathway were significantly upregulated, suggesting that chronic inflammation may be activated in sarcopenic patients; key precursor substances (such as 1,4-androstadiene-3,17-dione) in the steroid hormone synthesis pathway were significantly downregulated, indicating that hormone synthesis function may be impaired. The disorder of these pathways may synergistically promote the occurrence and development of sarcopenia.

#### 3.4.2 Correlation analysis of differential metabolites

**Figure 6:**
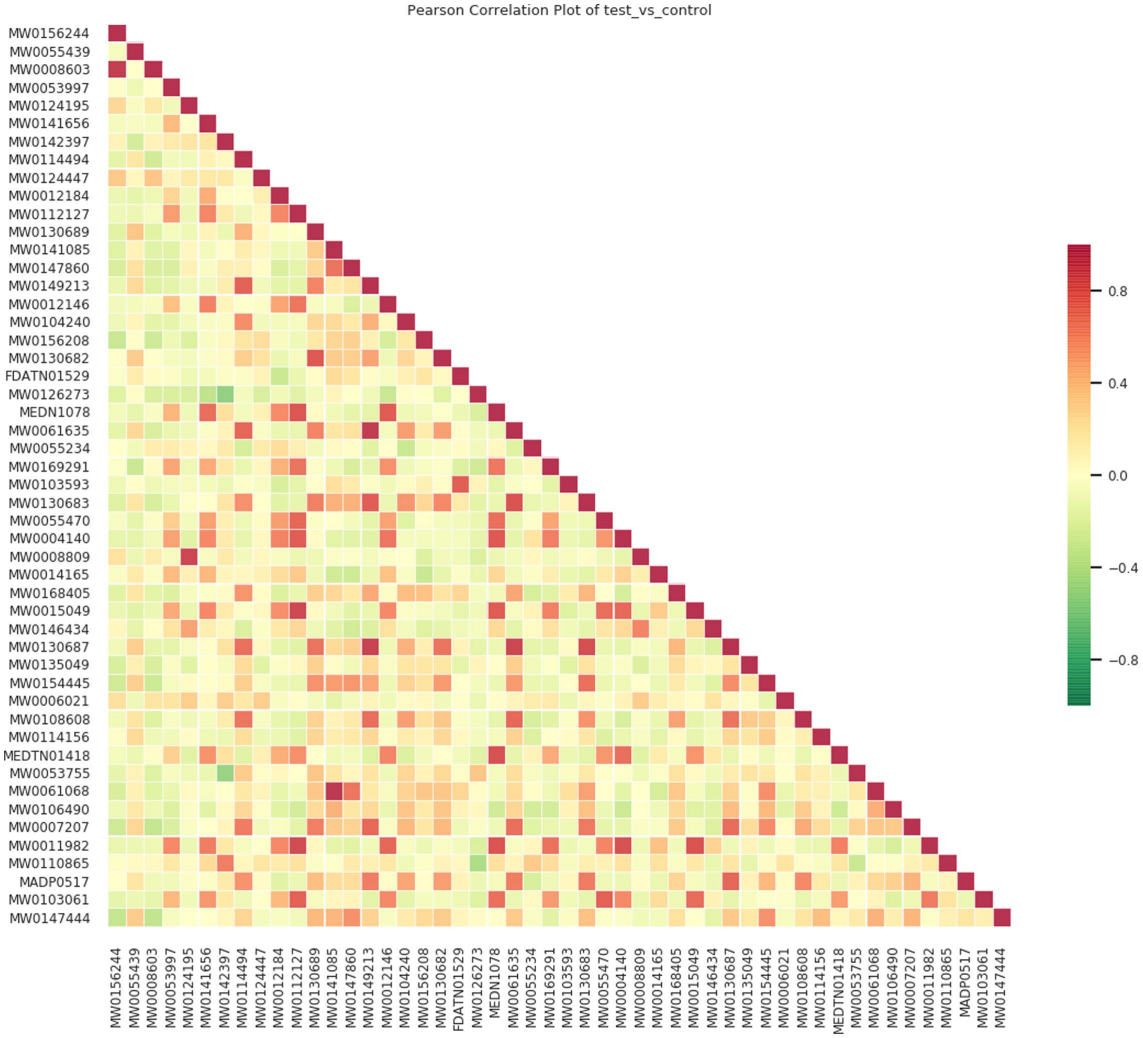
Correlation heatmap of differential metabolites. The horizontal and vertical axes are the names of differential metabolites; different colors represent the level of Pearson correlation coefficient. The relationship between the correlation coefficient and color is shown in the legend on the right. Red indicates a strong positive correlation, green indicates a strong negative correlation, and the darker the color, the greater the absolute value of the correlation coefficient between samples. By default, all differential metabolites are plotted. When the number of differential metabolites exceeds 50, the top 50 differential metabolites with the largest VIP values are displayed.

**Figure 7:**
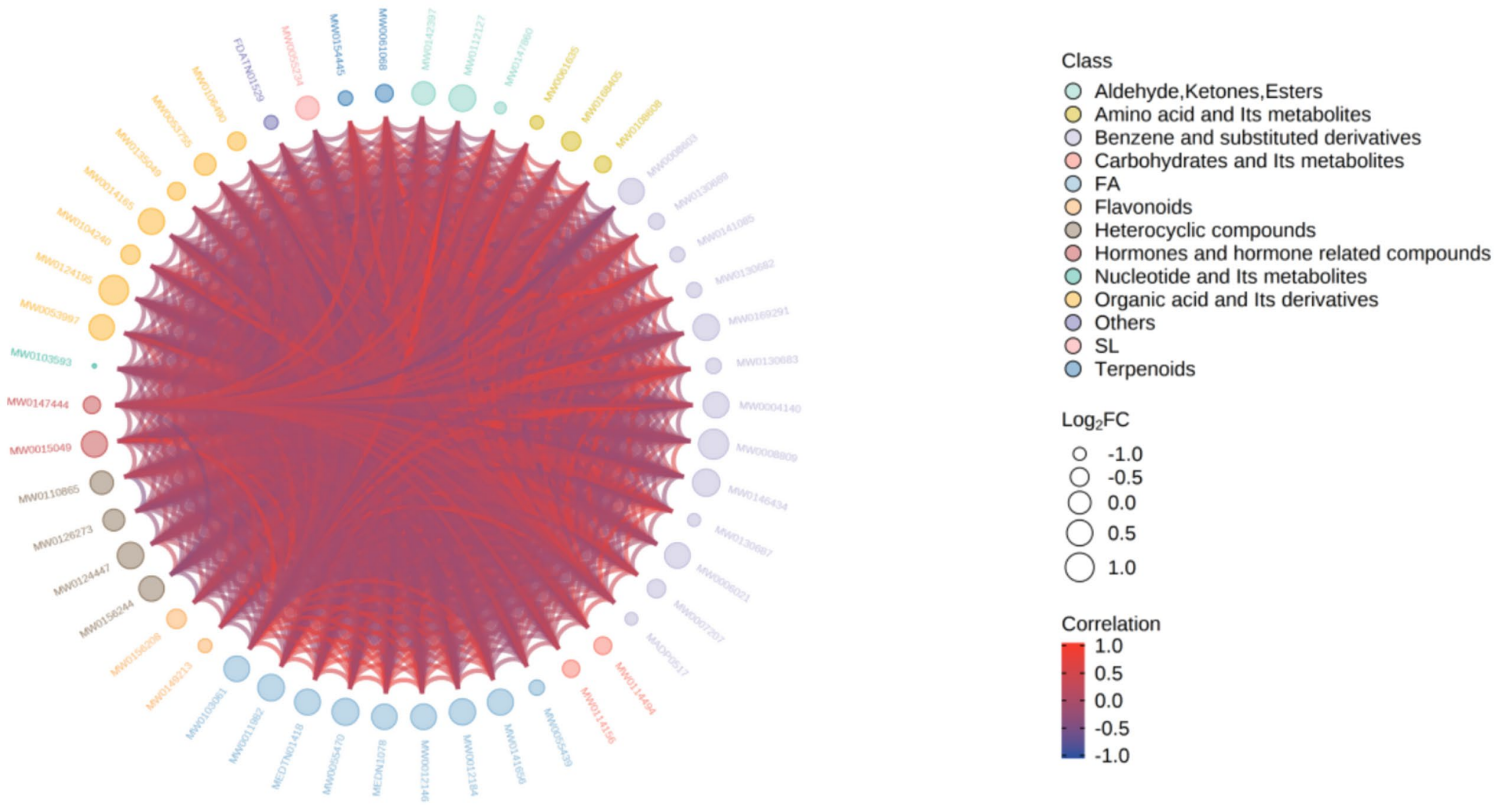
Chord diagram of differential metabolites. The outermost layer of the figure is the name of the differential metabolite, and the size of the dot represents the log FC value of the corresponding differential metabolite; different colors represent different classifications (class) of the corresponding differential metabolite; the connection line represents the Pearson correlation coefficient between the corresponding differential metabolites. The red line represents a positive correlation, and the blue line represents a negative correlation. By default, all differential metabolites are plotted. When the number of differential metabolites exceeds 50, the top 50 differential metabolites with the largest VIP values are displayed.

**Figure 8:**
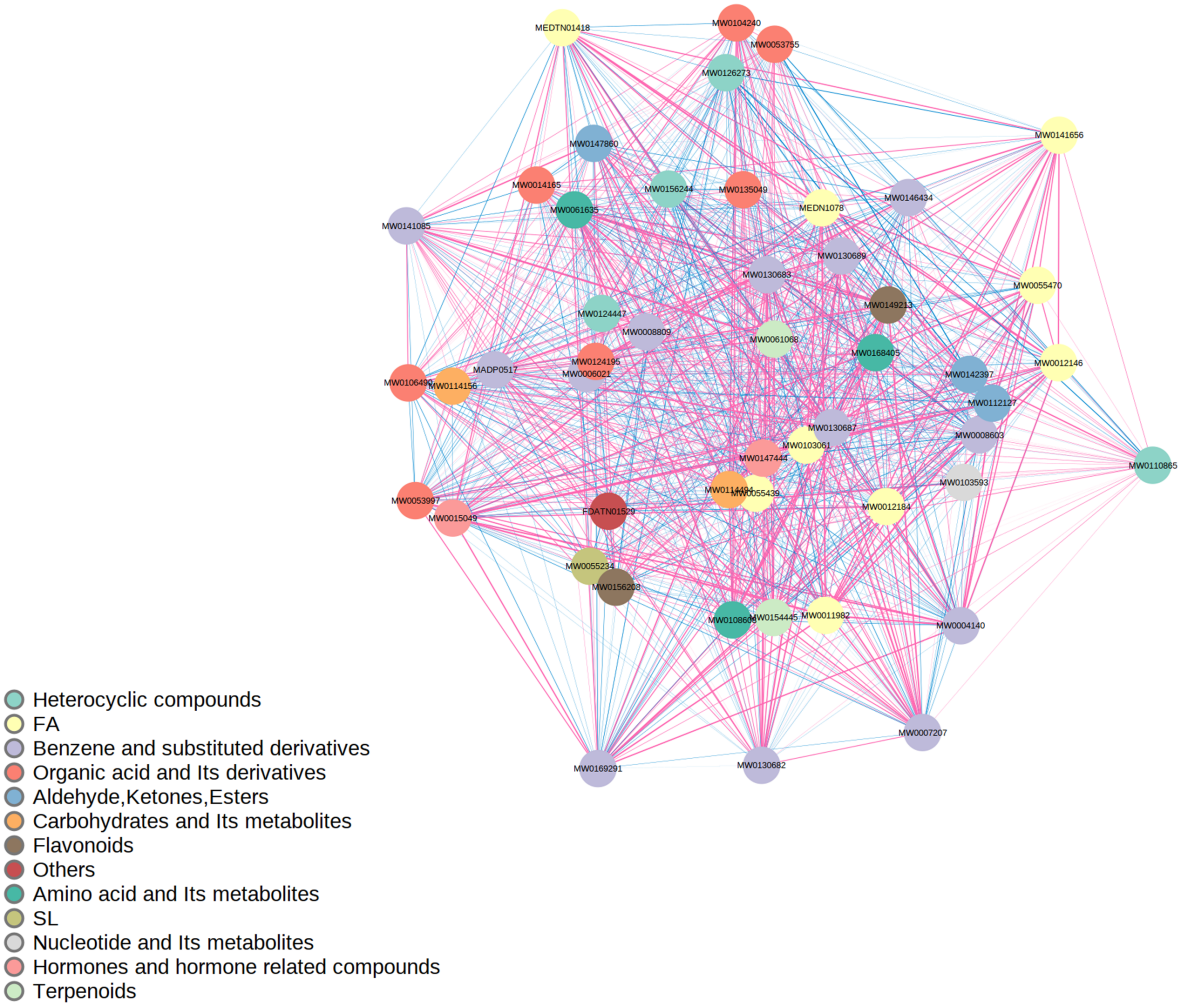
Correlation network diagram of differential metabolites. The dots in the figure represent significantly differential metabolites, and the size of the dot is related to the degree of connectivity. The larger the connectivity, the larger the dot, that is, the more dots (neighbors) connected to it. The pink line represents a positive correlation, and the blue line represents a negative correlation. The thickness of the line represents the absolute value of the correlation coefficient. The thicker the line, the greater the correlation. By default, all differential metabolites are plotted. When the number of differential metabolites exceeds 50, the top 50 differential metabolites with the largest VIP values are displayed.

Pearson correlation coefficient was used to analyze the association between 203 differential metabolites, and a correlation heatmap (Figure 6), correlation network diagram, and chord diagram (Figures 7, 8) were drawn. The results showed that there were complex synergistic or antagonistic relationships between differential metabolites: inflammation-related metabolites (such as 9S-HOTrE) were negatively correlated with amino acid metabolites (such as D-glutamic acid), suggesting that inflammatory responses may exacerbate muscle metabolic disorders; metabolites related to steroid hormone synthesis were positively correlated, indicating that the regulation of the hormone synthesis pathway was synergistic. These correlation relationships provide important evidence for revealing the metabolic regulatory network of sarcopenia.

## 4 Discussion

This study adopted an untargeted metabolomic method based on UPLC-MS/MS technology to explore the plasma metabolic profile differences between sarcopenic and non-sarcopenic older patients with sepsis. Among the 4,752 identified metabolites, 203 differential metabolites were screened out, including 71 upregulated and 132 downregulated ones, indicating that the plasma metabolic profile of sarcopenic patients presents a significant “inhibitory” feature. To control for the potential effects of age, BMI, and ADL on metabolic phenotypes, metabolic differences between groups remained significant after adjustment by multivariate analysis of covariance (ANCOVA), indicating that the relevant metabolic characteristics were primarily attributed to sarcopenia itself rather than the aforementioned confounding factors. These differential metabolites may be associated with and involved in multiple links such as the imbalance of muscle synthesis and decomposition, energy supply disorders, and impaired injury repair, collectively forming the potential metabolic background for progressive loss of muscle mass and function.

In terms of anabolism, the downregulation of steroid hormones and their precursors represented by 1,4-androstadiene-3,17-dione indicates a general decrease in anabolic capacity (4, 11, 12, 20, 21). Specifically, 1,4-androstadiene-3,17-dione, as a key precursor for androgen synthesis, its deficiency will limit testosterone production, thereby inhibiting protein synthesis mediated by the Akt/mTOR signaling pathway (13) and weakening the inhibitory effect on the ubiquitin-proteasome system (22), leading to myofiber atrophy. Previous studies have also shown that the downregulation of the steroid hormone pathway is closely related to sarcopenia (1–4, 20). For example, in elderly patients with chronic diseases, the decrease in androgen levels has been confirmed to be associated with the decline in muscle mass and function (2, 4). In addition, as a substrate for protein synthesis, a precursor of glutathione, and an intermediate of the tricarboxylic acid cycle, the deficiency of D-glutamic acid limits protein synthesis, impairs antioxidant capacity, and affects energy supply (11, 12). The reduction of isobutyric acid further leads to decreased muscle mass and reduced energy metabolism efficiency by weakening β-oxidation energy supply and the activation of the mTOR pathway by branched-chain amino acids (13–15). This is consistent with previous studies on amino acid metabolic disorders in sepsis patients (5–7, 10). Changes in these metabolites may synergistically lead to muscle anabolic resistance and break the balance between synthesis and decomposition.

In terms of catabolism, the upregulated metabolites 9S-HOTrE and L-arabitol are involved in inflammation, energy metabolism disorders, and oxidative stress, respectively. 9S-HOTrE synchronously upregulates the protein degradation system and pro-inflammatory factors by activating the NF-κB pathway (11, 23–26), inhibits the expression of myogenic differentiation factors such as MyoD and myogenin, impairs the regenerative potential of satellite cells (23, 27–29), and may interfere with sarcoplasmic reticulum calcium ion handling, damaging muscle regeneration and contractile function. This finding is consistent with previous studies. For example, in sepsis-induced muscle atrophy models, the activation of the arachidonic acid metabolic pathway has been shown to promote inflammatory responses and muscle decomposition (7, 11, 30). At the same time, the accumulation of L-arabitol suggests mitochondrial dysfunction and glycolytic compensation (13, 31), which is consistent with the characteristics of energy metabolism disorders in elderly sarcopenia (12).

The above metabolic changes of synthetic inhibition and enhanced decomposition are not isolated but form an interacting pathological network. Further KEGG pathway analysis confirmed the core status of pathways such as “branched-chain amino acid degradation, arachidonic acid metabolism, and steroid hormone synthesis” (3, 4, 8, 11–13, 20, 30). On the one hand, the activity of branched-chain amino acid transaminase and dehydrogenase in the skeletal muscle of elderly sarcopenic patients is increased, leading to the excessive decomposition of branched-chain amino acids into keto acids, while the utilization rate during muscle protein synthesis is significantly reduced. Among them, the key role of leucine in activating the mTORC1 pathway is also blocked, resulting in insufficient net protein synthesis, showing the characteristics of “enhanced decomposition but insufficient synthetic utilization” (13), which is similar to the metabolomic research results of sarcopenia in healthy elderly people (12–15). On the other hand, the activation of arachidonic acid metabolism produces a large number of mediators such as prostaglandins and leukotrienes, which promote protein degradation and inhibit insulin sensitivity by activating the NF-κB and p38 MAPK pathways (30), making arachidonic acid metabolic disorders an important source of chronic inflammation in sarcopenia (8, 11, 24, 25, 30). The downregulation of the steroid hormone synthesis pathway directly leads to the deficiency of protective hormones such as androgens, making muscles lose both anabolic drive and anti-inflammatory barrier support (2–4, 20). On the other hand, energy metabolism also presents the characteristics of “mitochondrial dysfunction and glycolytic compensation” (13, 20, 32). The accumulation of mitochondrial DNA mutations and the decrease in respiratory chain complex activity in skeletal muscle lead to reduced oxidative phosphorylation efficiency (13, 20, 32). To compensate for the energy gap, the expression of key glycolytic enzymes is upregulated, but the accumulation of anaerobic metabolites further aggravates metabolic disorders (11–13).

Placing this study in a broader research context, our findings have interesting intersections with recent studies on sepsis metabolic typing. For example, Alipanah-Lechner et al. classified acute respiratory distress syndrome (ARDS) into high-inflammatory and low-inflammatory subtypes and found that patients with the high-inflammatory subtype also showed significant downregulation of lipid metabolism and upregulation of glycolysis (7, 9). This partially overlaps with the metabolic characteristics observed in our sarcopenia group, especially those patients who may be in a higher inflammatory state, suggesting that there may be a common “high inflammation-metabolic disorder” phenotype in sepsis complications of different organ systems (5, 7, 23–25, 30).

This study has the following limitations: Firstly, this is a single-center, cross-sectional observational study with a relatively limited sample size, which only reveals the statistical association between metabolites and sarcopenia status rather than a causal relationship. Whether the observed “inhibitory” metabolic characteristics are the cause or consequence of sepsis-related sarcopenia needs to be verified in future multi-center, large-sample studies, and longitudinal cohort studies should be conducted to clarify their dynamic evolution rules and prognostic value. Secondly, the focus of this study is to reveal the metabolic differences between sarcopenic and non-sarcopenic subgroups within the sepsis population. Due to the lack of control groups such as “sarcopenia without sepsis” in the study design, we cannot currently accurately distinguish to what extent the observed metabolic changes are “sepsis-specific,” “sarcopenia-common,” or “sepsis-related sarcopenia-specific.” This is an important limitation of this study and a direction that needs to be improved in future research. Thirdly, metabolomic analysis suggests potential pathophysiological mechanisms, but this study did not experimentally verify the biological functions of these key metabolites at the cellular or animal model level. Future work needs to combine basic experimental research to clarify their specific mechanisms of action.

## 5 Conclusion

Through untargeted metabolomic analysis, this study preliminarily reveals the unique plasma metabolic profile of elderly patients with sepsis complicated by sarcopenia, which is characterized by the impact on core pathways such as branched-chain amino acid degradation, steroid hormone synthesis inhibition, and arachidonic acid metabolism activation. These findings systematically depict the potential metabolic imbalance networks associated with this disease state, provide new clues for understanding its pathophysiological mechanisms, and offer an important theoretical basis and potential targets for the future development of early diagnostic biomarkers and targeted intervention strategies for this disease.

## Data Availability

The original contributions presented in the study are included in the article/Supplementary material, further inquiries can be directed to the corresponding author.
