# Supplementary material for: Metabolomic biomarker differences of sarcopenia in older patients with sepsis
Source: Front Med (Lausanne). 2026 Apr 30;13:1797866. doi: 10.3389/fmed.2026.1797866 (PMC13171395; doi:10.3389/fmed.2026.1797866)
Supplement: Supplementary file 1 [file Data_Sheet_1.DOCX]

Supplementary Table 1: Stability of Internal Standards in QC Samples

| Q1 (Da) | RT (min) | CV |
| --- | --- | --- |
| 407.2792 | 6.23 | 0.0523 |
| 195.0504 | 0.80 | 0.0648 |
| 126.1457 | 5.09 | 0.0694 |
| 144.1115 | 6.26 | 0.0743 |

Supplementary Table 2: Statistics of Differential Metabolites Annotated by Each KEGG Pathway

| ko_ID | sig_compound | compound | sig_compound_all | compound_all |
| --- | --- | --- | --- | --- |
| ko01100 | 30 | 507 | 39 | 698 |
| ko04974 | 2 | 24 | 39 | 698 |
| ko00040 | 1 | 15 | 39 | 698 |
| ko00470 | 1 | 22 | 39 | 698 |
| ko00564 | 2 | 42 | 39 | 698 |
| ko00590 | 3 | 29 | 39 | 698 |
| ko00591 | 1 | 26 | 39 | 698 |
| ko00592 | 1 | 22 | 39 | 698 |
| ko04723 | 1 | 29 | 39 | 698 |
| ko05231 | 2 | 36 | 39 | 698 |
| ko00052 | 1 | 9 | 39 | 698 |
| ko00140 | 1 | 37 | 39 | 698 |
| ko04114 | 1 | 3 | 39 | 698 |
| ko04913 | 2 | 7 | 39 | 698 |
| ko04914 | 1 | 2 | 39 | 698 |


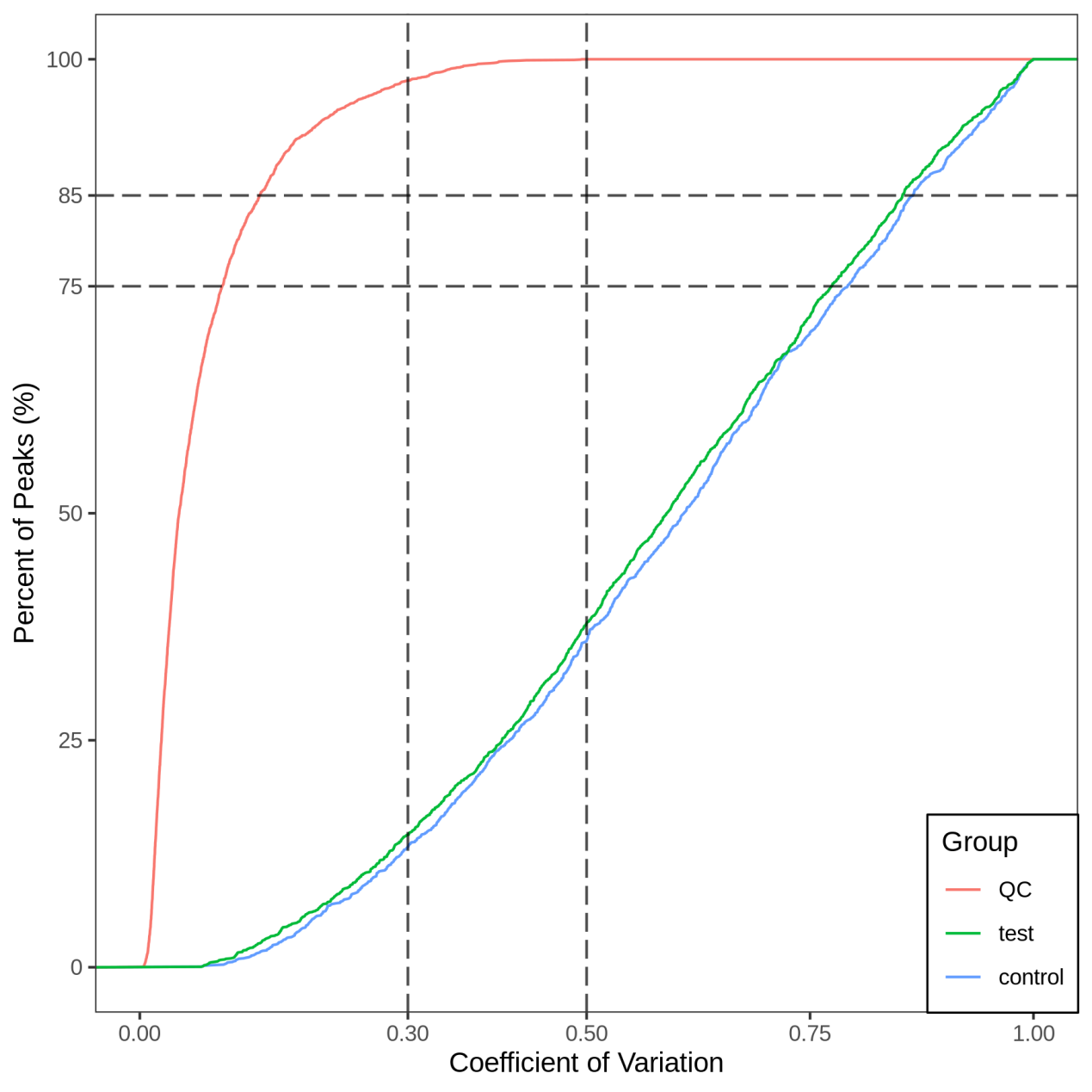


**Supplementary Figure 1: CV Distribution of Samples in Each Group**

(Note: The abscissa represents CV values, and the ordinate represents the proportion of substances with CV values less than the corresponding value to the total number of substances. The same color represents samples from the same group, and QC refers to quality control samples. The two vertical reference lines corresponding to the X-axis have CV values of 0.3 and 0.5, and the two horizontal reference lines corresponding to the X-axis correspond to 75% and 85% of the total number of substances.)


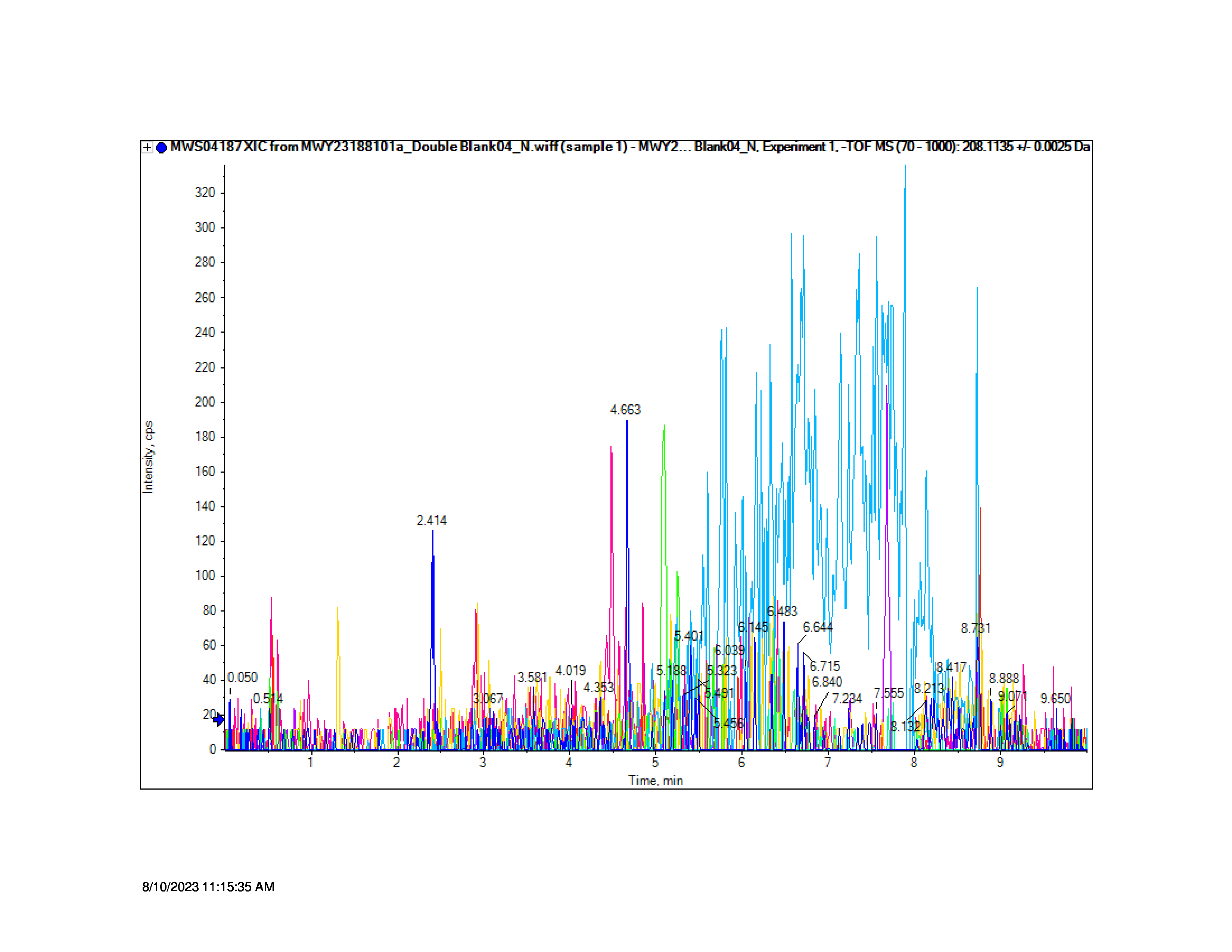


**Supplementary Figure 2 : EIC of Internal Standards in Blank Samples**

(Note: All signals in the above EIC charts are noise peaks, and there is no obvious signal peak of the internal standard substance at the corresponding time.)


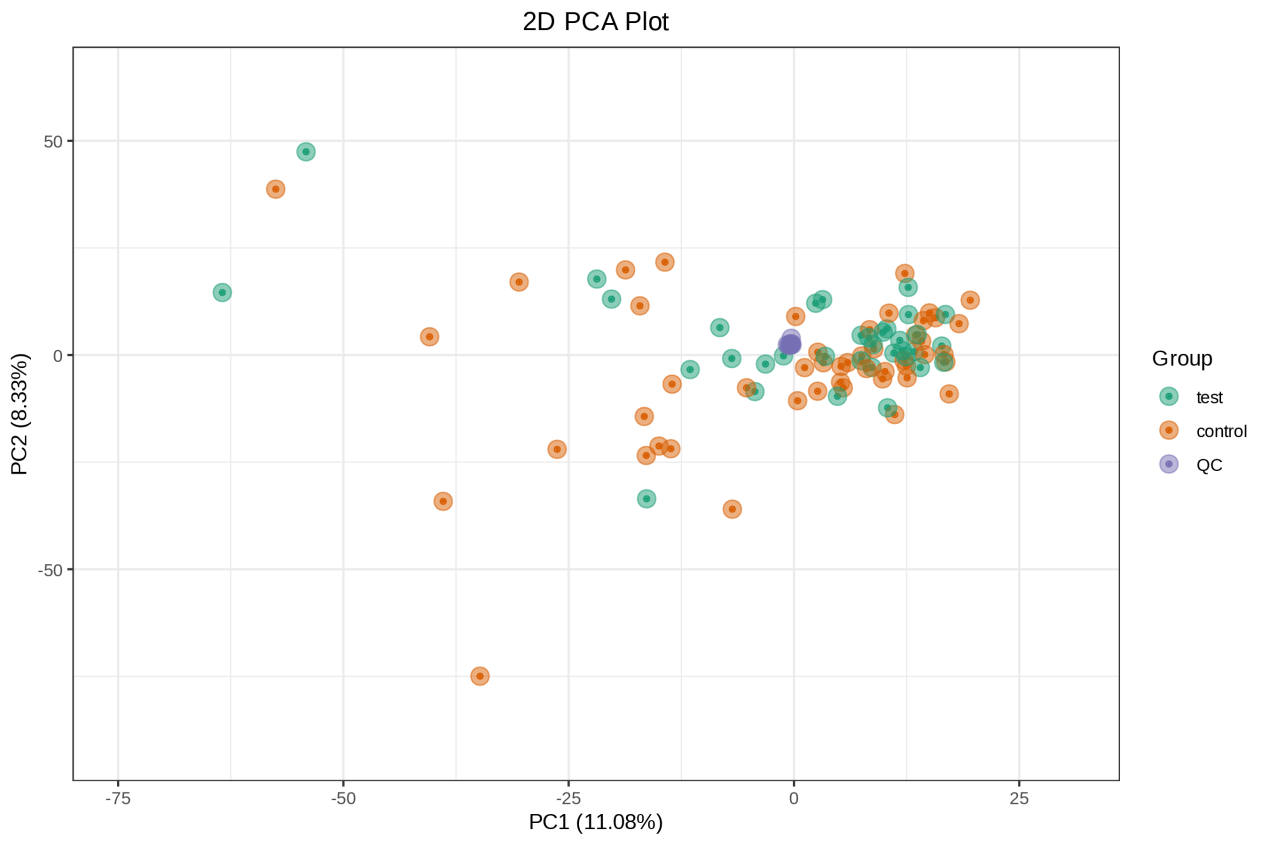


**Supplementary Figure 3: PCA Score Plot of Mass Spectrometry Data of Samples in Each Group and Quality Control Samples**

(Note: PC1 represents the first principal component, PC2 represents the second principal component, PC3 represents the third principal component, and the percentage represents the explanation rate of the principal component to the data set; each point in the figure represents a sample, samples from the same group are indicated by the same color, and Group represents the grouping.)


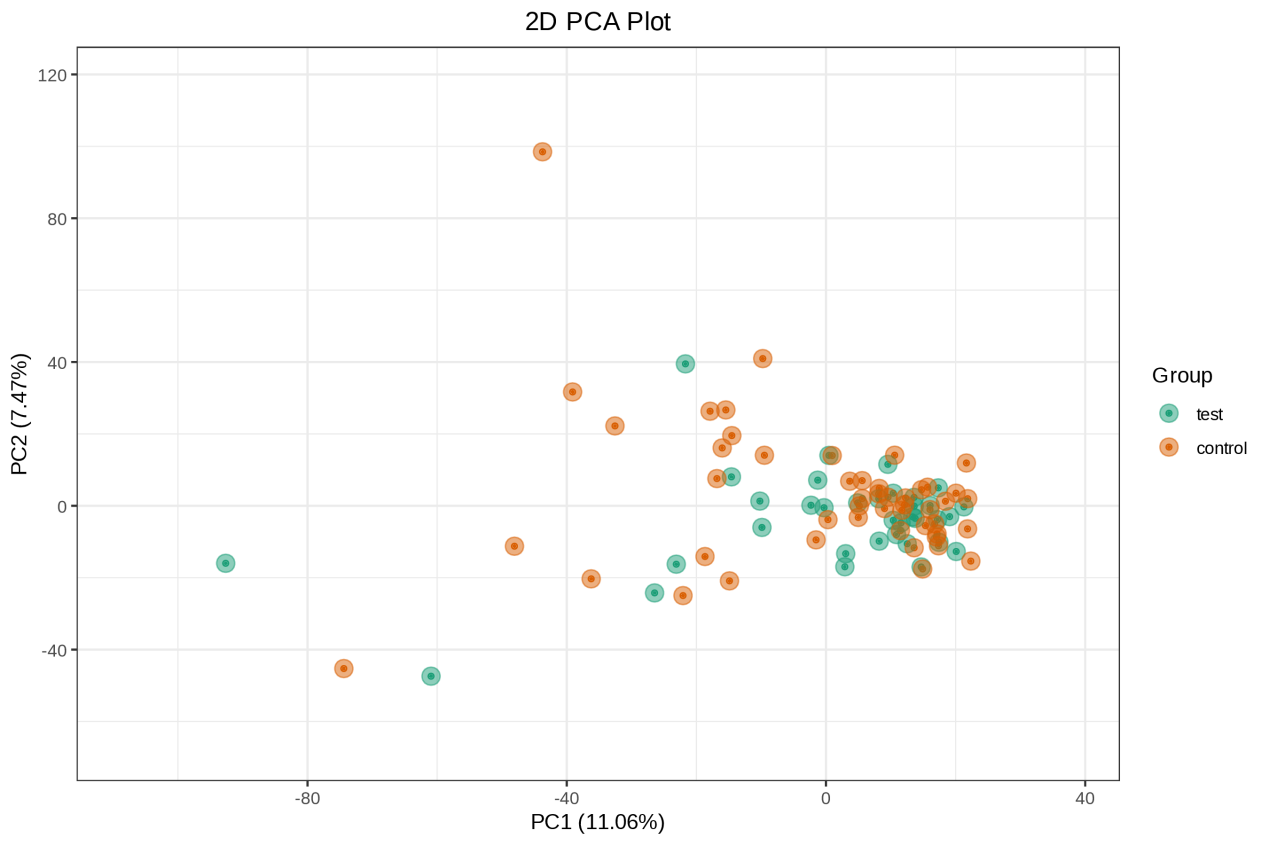


**Supplementary Figure 4: Grouped Principal Component Analysis Plot**

(Note: Each PCA plot corresponds to a different grouping. PC1 represents the first principal component, PC2 represents the second principal component, and the percentage represents the variance explanation rate of the principal component to the data set; each point in the figure represents a sample, samples from the same group are indicated by the same color, and Group represents the grouping.)


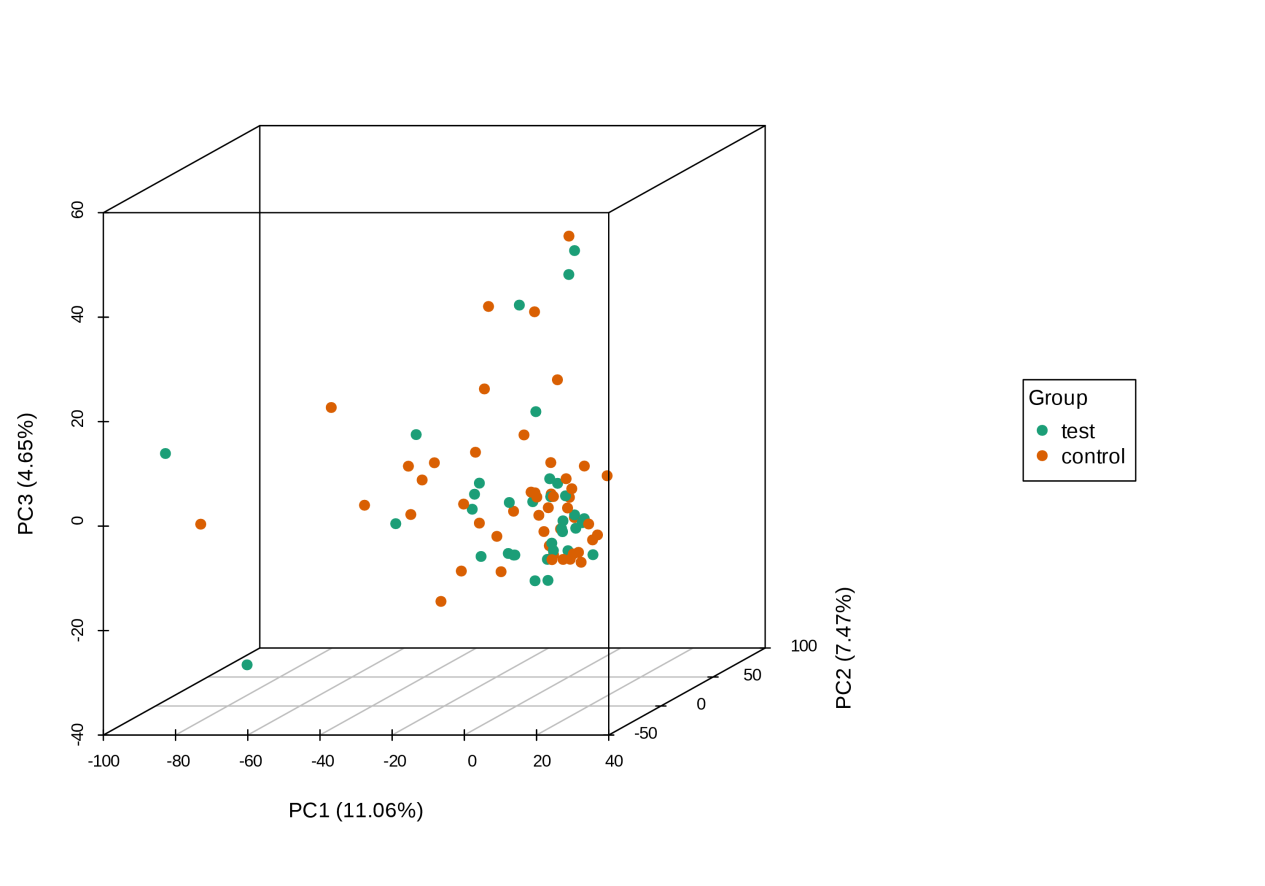


**Supplementary Figure 5: Three-Dimensional Plot of Grouped Principal Component Analysis**

(Note: PC1 represents the first principal component, PC2 represents the second principal component, and PC3 represents the third principal component.)


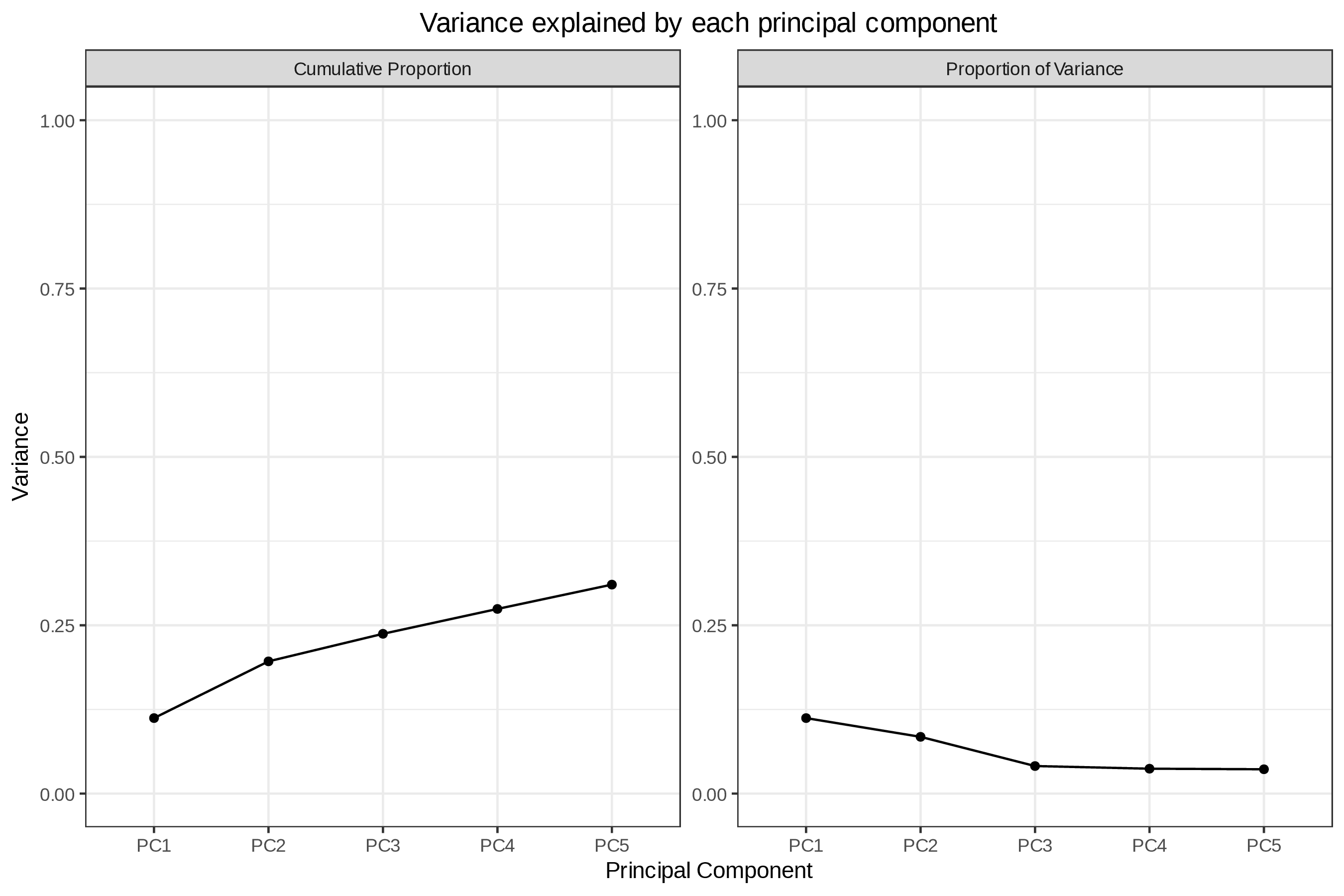


**Supplementary Figure 6: Explanation Rate Plot of Grouped Principal Component Analysis**

(Note: The abscissa represents each principal component, and the ordinate represents the explanation rate to the data set. The left figure is the cumulative explanation rate, and the right figure is the explanation rate of each principal component.)


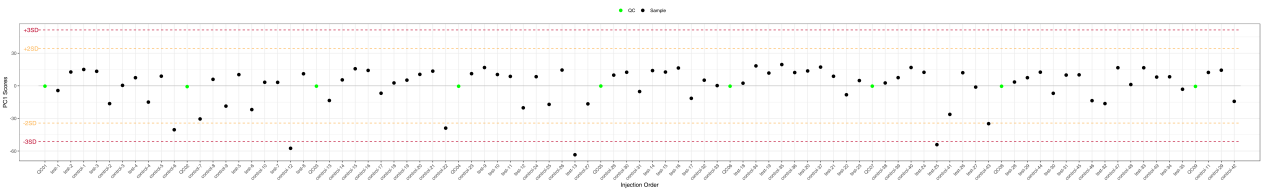


**Supplementary Figure 7: Overall Sample PC1 Control Chart**

(Note: The abscissa in the figure is the sample detection order, and the ordinate reflects the PC1 value. The yellow and red lines define the ranges of ±2 and ±3 standard deviations, respectively. Green points represent quality control (QC) samples, and black points represent test samples.)


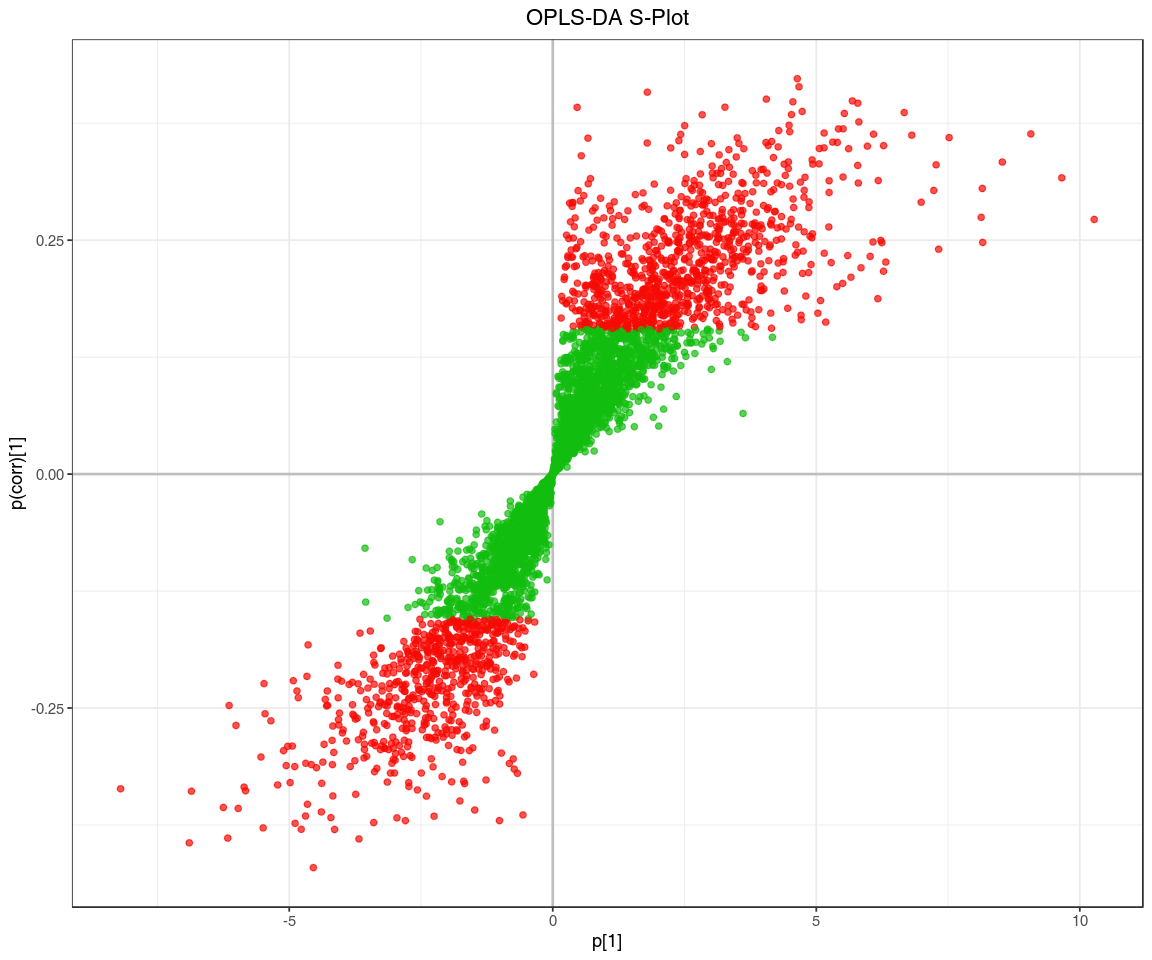


**Supplementary Figure 8: OPLS-DA S-Plot**

(Note: The abscissa represents the covariance between the principal component and metabolites, and the ordinate represents the correlation coefficient between the principal component and metabolites. Metabolites closer to the upper right and lower left corners indicate more significant differences. Red points indicate that the VIP value of these metabolites is greater than 1, and green points indicate that the VIP value of these metabolites is less than or equal to 1.)


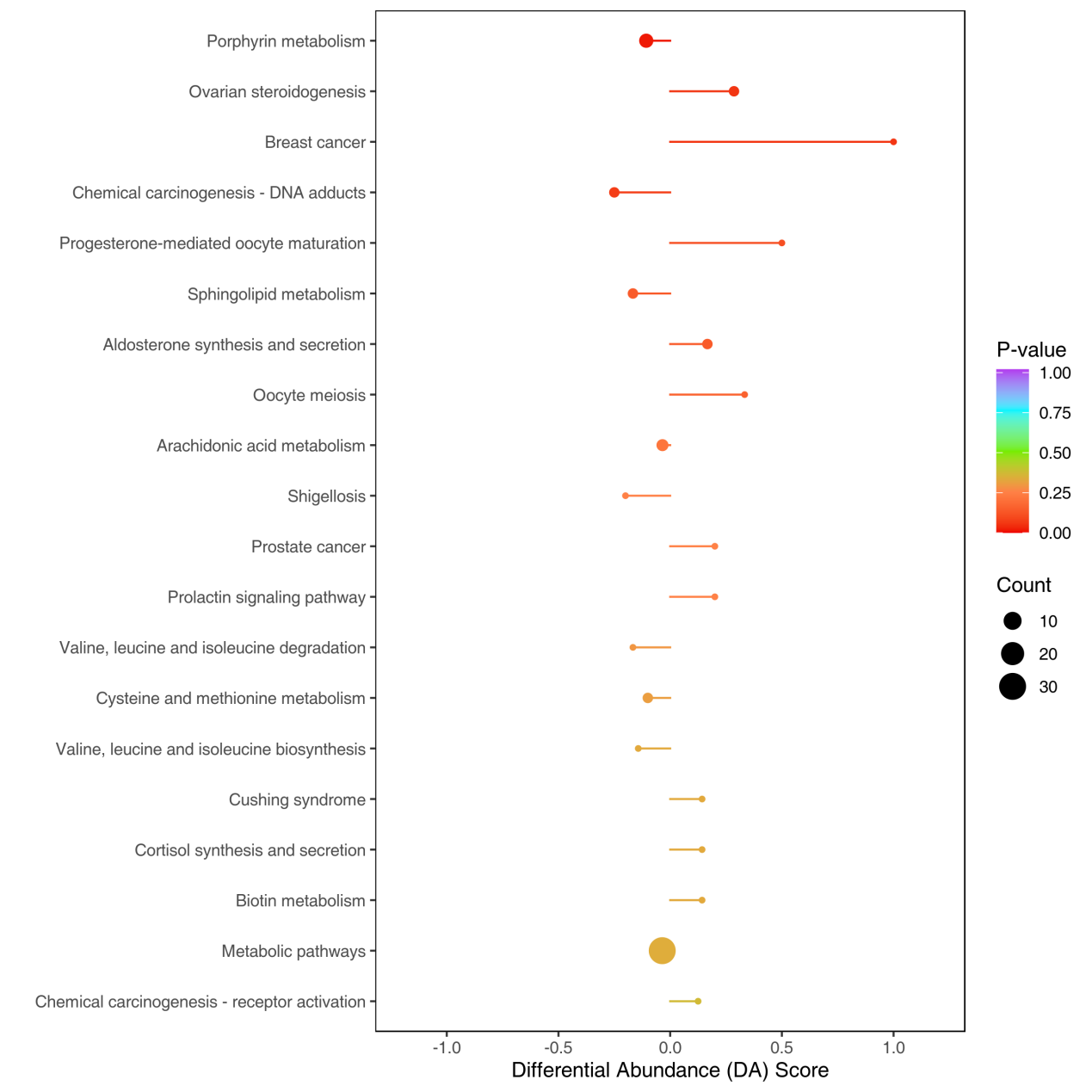


**Supplementary Figure 9: Differential Abundance Score Plot**

(Note: The ordinate represents the name of the differential pathway (sorted by P-value), and the abscissa represents the Differential Abundance Score (DA Score). DA Score reflects the overall change of all metabolites in the metabolic pathway. A score of 1 indicates that all identified metabolites in the pathway show an upward expression trend, and a score of -1 indicates that all identified metabolites in the pathway show a downward expression trend. The length of the line represents the absolute value of the DA Score, and the size of the dot at the end of the line represents the number of differential metabolites in the pathway. Dots distributed on the left side of the central axis with longer lines indicate that the overall expression of the pathway tends to be more downregulated; dots distributed on the right side of the central axis with longer lines indicate that the overall expression of the pathway tends to be more upregulated; larger dots indicate a greater number of metabolites. The color of the lines and dots reflects the P-value: the closer to red, the smaller the P-value; the closer to purple, the larger the P-value.)


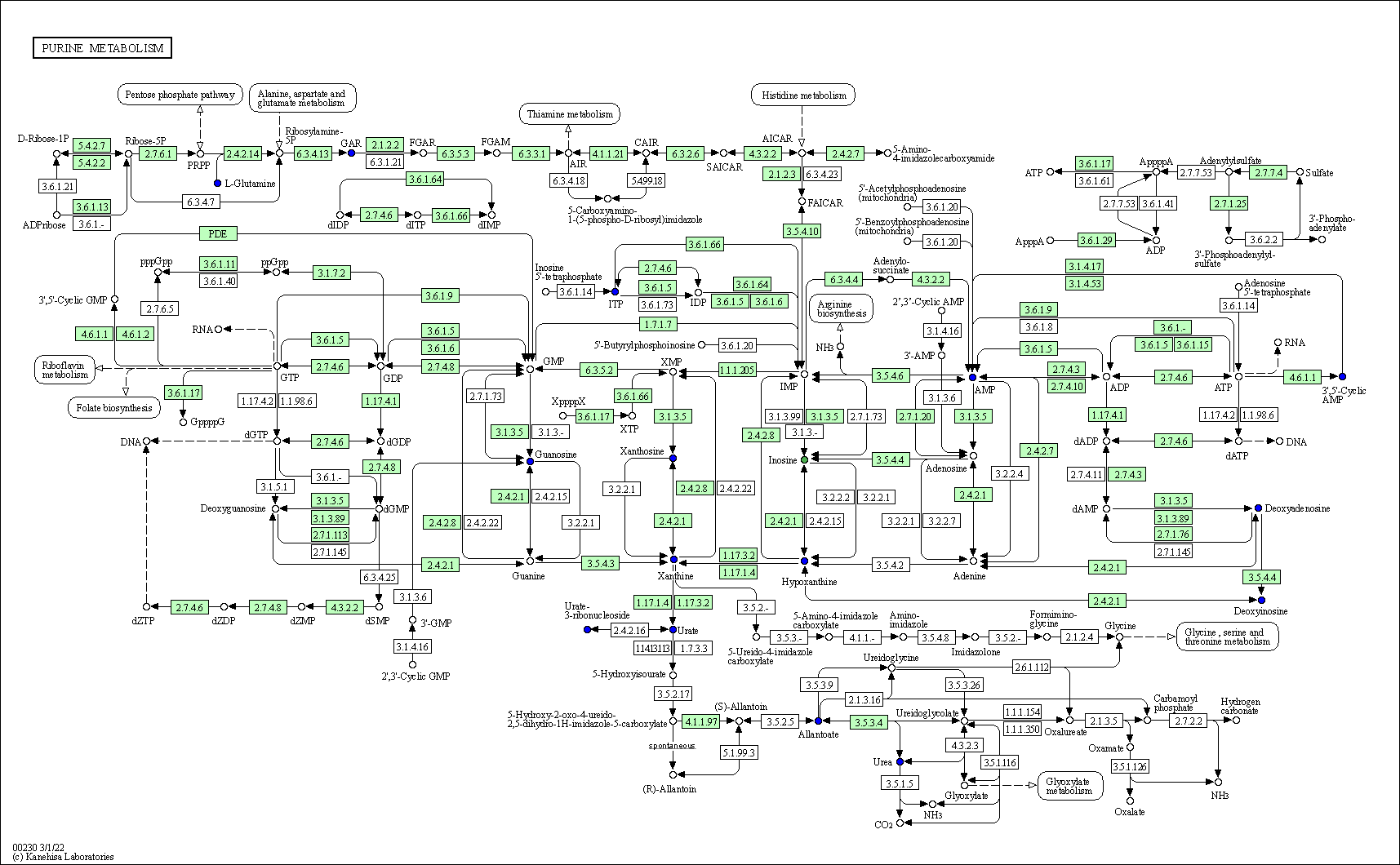


**Supplementary Figure 10: KEGG Pathway Map of Differential Metabolites**

(Note: Red indicates that the metabolite content is significantly upregulated in the experimental group, blue indicates that the metabolite is detected but no significant change occurs, green indicates that the metabolite content is significantly downregulated in the experimental group, and orange indicates that it contains both upregulated and downregulated metabolites. The causes of phenotypic differences in the research objects are explored through metabolic pathways.)
